# PHL13/484: A Systematic Review on the Use of Interactive Communication for Consumer Health Education and the Impact of the Internet on Public Health

**DOI:** 10.2196/jmir.1.suppl1.e93

**Published:** 1999-09-19

**Authors:** G Eysenbach, ER Sa, D O'Connor, A Jadad

**Affiliations:** ^1^Institute for Clinical Social Medicine, HeidelbergGermany

**Keywords:** Evidence-Based Medicine, Systematic Reviews, Public Health, Patient-Physician Relationship

## Abstract

**Introduction:**

In an ongoing systematic review of the Cochrane Consumers & Communication Group we are compiling evidence on the impact of the Internet on public health. We include any "interactive health communication", i.e. any electronic interaction of an individual--consumer, patient, caregiver, or professional-with an electronic device or communication technology to access or transmit health information or to receive guidance on a health-related issue.

**Methods:**

We will systematically search for and compile in an overview evidence for the effectiveness and impact of interactive communication for consumer health education. Evidence here means studies such as randomised controlled trials (RCTs), controlled clinical trials (CCTs), controlled before and after (CBA) studies, interrupted time series (ITS) studies. Interventions to be compared against each other include a) consumers having been exposed to Internet-based health information against consumers having been exposed to traditional ways of health information, such as face-to-face interpersonal interactions, courses, printed material, self-help groups etc., and b) consumers having been exposed to Internet-based education against consumers having been exposed to Internet-based education using a different approach/method. As outcome measures we are especially looking for studies determining, a change of health related knowledge, attitude and behavior of participants, a change of health status of participants, the satisfaction of participants and health professionals and the change of relationship between patients and health professionals.

**Results:**

Up to now, only one RCT has been found, highlighting the urgent need for more sound studies to determine the impact of the Internet on Public Health.

**Discussion:**

The systematic review is still in progress and we invite authors of published and unpublished research addressing these questions to submit their work to us for inclusion into the analysis.

